# Targeting Polymorphonuclear Leukocytes in Acute Myocardial Infarction

**DOI:** 10.1100/tsw.2007.45

**Published:** 2007-02-02

**Authors:** Clara Di Filippo, Francesco Rossi, Michele D'Amico

**Affiliations:** ^1^ Department of Experimental Medicine, Section of Pharmacology “L. Donatelli”, 2nd University of Naples, Naples, Italy; ^2^ Excellence Centre for Cardiovascular Diseases, 2nd University of Naples, Naples, Italy

**Keywords:** acute myocardial infarct, polymorphonuclear leukocytes, oxidative stress, inflammation, phosphoinositide 3-kinase

## Abstract

Several studies have recognized the strong impact that the acute myocardial infarctions (AMI) have on the morbidity and mortality of patients affected by cardiovascular diseases. Still open, however, is the field concerning the mediators and the pathways involved in the etiology of this cardiovascular event. The present review would support the relatively new discovered role that the polymorphonuclear leukocytes (PMNs) have in the pathogenesis of the AMI, through a brief analysis of past and ongoing research. Particularly, it is reviewed here the possibility that inhibition of the activity of PMNs and inhibition of the signaling pathways related to their activity may result useful in AMI and may improve the prognosis of this pathology. This review, indeed, presents and discusses new data on one of the lipid kinase, the phosphoinositide 3-kinase gamma (PI3Kγ), and its role in neutrophil recruitment during AMI.

## INTRODUCTION

Cardiovascular pathologies are many and important. They include thrombotic stroke, embolic vascular occlusions, angina pectoris, peripheral vascular insufficiency, cardiac surgery, organ transplantation, and cardiogenic shock[1]. Among these acute myocardial infarction (AMI) is one of the most frequent and mortal today. AMI is a circumstance characterized by two events: the ischemia and the reperfusion of the myocardium, leading to injury of the myocardium and loss of its function. Although in patients with AMI, early reperfusion enhances structural and functional recovery of the myocardium and improves survival[2], accumulating experimental evidence has indicated that myocardial reperfusion can promote potentially cardiotoxic inflammatory reactions, eg, cytokine production, fever, complement activation, leukocytosis, acute-phase protein synthesis, and tissue polymorphonuclear (PMNs) leukocytes infiltration at last. These latter can accumulate in ischemic-reperfused myocardium and can have cardiotoxic effects as an important biological source of oxygen free radicals.

The major objective of this review is to briefly examine the ongoing research on the hypothesis that inhibition of the PMNs and inhibition/stimulation of the signalling pathways related to their activity may result useful in AMI. Even if further work is required to define the precise role of PMNs and their relative importance in the pathophysiological processes occurring at various times during myocardial ischemia and reperfusion this review would provide a rationale for designing clinically applicable interventions directed at inhibiting PMN-mediated myocardial damage.

## The PMN Cell

PMNs are usually thought of as the leukocyte population involved in acute inflammatory responses, acting as a first line of defence against invading microorganisms. These terminally differentiated cells are generally not thought of as an important source of de novo synthesis of polypeptide mediators. Recent progress has shown, however, that PMNs are able to synthesize cytokines in response to a variety of inflammatory stimuli and during certain pathological conditions. The expression profiles of PMN-derived cytokines are similar with those of monocytes/macrophages, major professional phagocytes. Like monocytes, PMNs are able to secrete proinflammatory cytokines [e.g., tumor necrosis factor (TNF)-alpha and interleukin (IL)-1beta], both CC and CXC chemokines [e.g., IL-8, interferon-inducible protein 10 (IP-10) and macrophage inflammatory protein (MIP)-1alpha], and angiogenic factors [e.g., vascular endothelial growth factor (VEGF)]. The secretion of cytokines by activated PMNs is regulated by immunoregulatory cytokines such as interferon (IFN)-gamma, IL-4, IL-10 and IL-13. In addition to acute inflammatory responses, PMNs and PMN-derived cytokines appear to be involved in the pathogenesis of such chronic inflammatory disorders as rheumatoid arthritis, inflammatory bowel diseases and mycobacterial infections.

## The PMN Interaction With Myocardial Ischemic Tissue

The recruitment of polymorphonuclear neutrophils in the ischemic heart involves PMN adhesion to the endothelial cells and migration into the tissue[3]. The PMNs adhesion includes the process of tethering, rolling, activation and firm adhesion[3,4].

Rolling and tethering of circulating PMNs to the endothelial cells involves the selectin family of adhesion molecules and exposure to various PMN activators[5]. Selectin- mediated events slow the velocity of the PMNs allowing endothelial-derived factors (i.e. platelet activating factors, interleukin- 8) to affect PMNs function[6]. The family consists of three closely related cell surface molecules: L-selectin, P-selectin and E-selectin.

L-selectin is constitutively expressed on the PMNs surface and it expression is decreased after PMNs activation by pro-inflammatory mediators such as interleukin-8 and PAF[7,8,9]. In contrast E-selectin is expressed only after activation of endothelial cells by cytokines such as interleukin-1 and TNF$\alpha$[10,11].

P-selectin is constitutively stored in platelets and endothelial cells; it is rapidly expressed on the endothelial surface after activation by inflammatory mediators such as histamine and thrombin[9,12]. Rolling and capture of PMNs are in large part mediated by endothelial P-selectin binding to P-selectin glycopotrein ligand-1 (PSGL-1) [13]. Experimental studies have suggested that monoclonal antibodies against L-selectin[14] and P-selectin were effective in reducing neutrophils accumulation into the heart thus reducing myocardial necrosis within the few hours after reperfusion[15,16]. However, other studies showed no difference in myocardial injury occurring during first 24h in P-selectin devoid mice, despite marked reduction of leukocyte accumulation[17,18].

Moreover selectin dependent-adhesion of leucocytes does not lead to firm adhesion and transmigration unless another group of adhesion molecules the so called integrin is involved [9]. Indeed, PMNs adhesion to the endothelium as well as PMN transendothelial migration are also mediated by β integrins molecules expressed on the surface of the activated neutrophils. Integrins are heterodimeric proteins with a common β subunit linked to a variable α subunit, neutrophils can express 13 different integrins among these $\beta_2$ sub-family repreents the most important in neutrophil-dependent inflammation[19,20]. The $\beta_2$ integrin sub-family consist of a common $\beta_2$ unit (CD18) linked to one of four α subunit indicated as CD11a, CD11b, CD11c or CD11d[21]. PMNs surface express constitutively $B_2$ integrins; proinflammatory or chemoattractant stimulus determinate up-regulation of CD11b/CD18 on the PMNs surface. This latter event is involved in PMNs attachment to the endothelium and their subsequent migration through endothelial cells[3,22,23,24].

The $\beta_{2-}$ integrin CD11a/CD18 and CD11b/CD18 appear the major negative regulators of PMN rolling velocity indeed deletion of $\beta_{2-}$ integrin mice (CD18$^{-/-}$mice) was shown to increase PMN rolling velocity. It has been shown by ourselves that myocardial infarction occurs in concomitance with increased CD11b expression on PMN cell surface[25], as well as ischemic preconditioning has been demonstrated to act through reduced neutrophil-endothelium interaction due to reduced expression of CD11b on PMN[26].

The endothelial ligand for neutrophils $\beta_2$ integrins is the intercellular adhesion molecule-1 (ICAM-1), responsible for firm adhesion of neutrophils to the endothelium. It is induced in heart after ischemia and reperfusion, and its expression not only promotes migration, stimulates production of superoxide, IL-8 and TNFα[27], but also gives basis to direct neutrophil-myocyte interaction and cytotoxicity[28]. Many studies have described that use of monoclonal ICAM-1[29,30,31,32] significantly attenuates myocardial ischemia reperfusion injury.

## The PMN Dependent Biochemical Scenario of The Myocardial Damage

Recruitment of activated neutrophils, with pro-inflammatory mediators, such as fMLP, C5a, PAF, TNF-α and interleukin-6, to the site of injury produces superoxide anion ($O_2^-$)[33,34,35]. $O_2^-$ is produced by activation of the PMN membrane-associated NADPH oxidase system that initiates a respiratory burst characterized by a marked increase in cellular oxygen consumption and generation of superoxide anions[36,37]. Although superoxide anion shows toxics effects by a direct interaction with cellular targets, it is wide documented that many dangerous tissue effects of $O_2^-$ result from the secondary formation of other oxygen radicals in addition to direct reactions of superoxide with biological targets, such as lipids[38,39], and DNA[40]. In fact superoxide undergoes a spontaneous dismutation reaction that yields one molecule each of $H_2O_2$ and oxygen. It is possible that hydrogen peroxide and superoxide anion may react to produce the most reactive oxy radical represented by $HO_2$ by a chemical process known as the Haber-Weiss reaction[41]. However, the enzyme myeloperoxidase (MPO) releases from azurophilic granules efficiently removes hydrogen peroxide by catalyzing the interaction of hydrogen peroxide with $Cl^-$ to form hypochlorous acid. Hypochlorous acid is a powerful oxidant that may chlorinate or oxidize a variety of target molecules, and reactions of hypochlorous acid with primary amines or ammonia can give rise to chloramines, which are also energetic oxidants[42].

Upon degranulation, activated PMNs mediate tissues damage also by release of proteolytic enzymes[43]. Serine and elastase proteinases[43,44]are highly positively charged, and their cationic nature may contribute to tissue damage by direct alterations in membrane charge distribution[34] or by enhancing binding to cell membranes and extra cellular matrix components[45]. Moreover elastase, increasing vascular permeability through vascular degrading basement, facilitates postischemic migration of PMNs[46,47]. Elastase is also contributor to the PMN mediated injury;it may act by hydrolyzing proteins of the extracellular matrix (eg, elastin, fibronectin, and collagen types III and IV) and plasma (eg, complement proteins and clotting factors)[44,48].

Elastase inhibitors have found to protect against PMNs-mediated cardiac ischaemia riperfusione injury[49,50].

Activated neutrophils also stimulate phospholipase $A_2$ generating 5-lipoxygenase products including leukotriene B4 (LTB4) and platelet-activating factor (PAF)[51,52]. Both of these stimulate PMNs chemotaxis, adhesion to endothelial cells, oxydative metabolism and degranulation[53,54] and they seem to amplify PMN-mediated tissue injury and vascular permeability[55]. In addition activated PMNs can release phospholipase $A_2$ into the external environment[56], enhancing production of eicosanoids and PAF by other cells[57,58]. It is to underline that LTB4, beyond its proinflammatory effects has important clinical actions during ischemia reperfusion, for example negative inotropic effects and induction of ventricular arrhythmias[59]. Moreover, in animal model also PAF, released by activated neutrophils, seems to contribute to coronary artery constriction, modulation of myocardial contractility as well as generation of arrhythmias during ischemic events. These, mediated by a direct action of PAF on cardiac tissues, or indirectly via the release of other mediators such as eicosonoids (Thromboxane A2, Leukotriene) or cytokines (TNF$\alpha$)[60,61,62].

## The Pharmacological Approach To PMN

Several conventional drugs used in the treatment of patients with myocardial ischemia (eg, calcium antagonists[63], lidocaine[64], heparin[65], captopril[66], angiotensin convertine enzyme inhibitors, angiotensin II type-1 receptor antagonists and statins[2] are shown to inhibit PMN function, in addition to their other pharmacological effects. However, the progress achieved in this field gives the right arm to the discovery of new tools and strategies. The first drugs to be mentioned are monoclonal antibodies directed towards adhesion molecules or their ligands (P-selectin, L-selectin, CD11b-CD18), and towards complement fragments or their receptors that attenuate neutrophil-mediated injury (vascular injury, infarction). Clinical application of these new agents has however limitations in the immunogenicity of the peptide reagents first used[31,67,68]. Humanized antibodies and non-peptide agents, such as oligosaccharide analogs to syalil Lewi-x (one of the main selectin ligands) may prove effective in this regard[69].

Both nitric oxide and adenosine, as regulators of blood flow and endothelial function, exhibit a wide range of effects against neutrophil-mediated events in myocardial ischemia/reperfusion. A human trial showed that adenosine, through its multi-targeted pharmacological actions, is able to limit accumulation of PMN, endothelial injury, and and infarct size[70]. Additional studies in patients with acute myocardial infarction have shown that adenosine treatment has been associated with decreases of the infarcts, decreases of no-reflow phenomenon and improved left ventricular function[71].

Acetaminophen with its antioxidant properties seems i) to inhibit the neutrophils functions[72], ii) to modulate the activities of myeloperoxidase[73], iii) to result cardioprotective in experimental animal model of myocardial ischemia reperfusion[74,75]. This is mostly evident in the preservation of contractile function, in the maintenance of myofibrillar integrity, and in the attenuation of damaging cardiac markers[76].

Also glucocorticoids are cardioprotectives in experimental ischemia reperfusion models. Particularly, dexametasone significantly reduced rat myocardial ischemia reperfusion injury, when given as pre-treatment, or at the beginning of the reperfusion period[25], thought this action partly exerted through annexin 1 (ANXA1). This 37-kDa protein has an N-terminal of 46 amino acids and it is expressed constitutively in many tissues and cells including lung, kidney, bone marrow, intestine, spleen, thymus, brain, neutrophils, monocyte/macrophage[77,78]. ANXA1 belongs to a family of $Ca^{2+}$ and phospholipid binding proteins of which 13 distinct members exist[79]. Structurally, these proteins are characterised by having a core of 4 or 8 conserved repeats each containing about 70 amino acids. The core is attached to an N-terminal segment that is unique for each member of the annexin family and it is thought to be responsible for each annexin's specific biological functions. Annexins proteins have been implicated in several biological functions ranging from membrane organization and exocytosis, cell growth and differentiation, ion channel formation, blood coagulation (annexin 5) and inhibition of inflammation (mainly annexin 1)[80,81,82,83].

At the beginning annexin-1 was defined as a glucocorticoid-inducible protein[84] that produced its anti-inflammatory effects by inhibiting phospholipase $A_2$ activity and thus preventing arachidonic acid mobilisation and prostanoid generation[85]. Further studies indicated that the full-length protein, retained anti-inflammatory in models insensitive to inhibitors of lipid metabolism[79,86,87]. Further work in addition to the development of Annexin 1 null mice [88] have allowed a better definition of the roles played by the endogenous protein in several cellular functions, including phagocytosis, extravasation, and mediator generation.

Promising are the studies conducted with exogenous recombinant annexin 1 and annexin1-derived N-terminus peptides annexin 1 derived peptides. Annexin 1 and annexin1-derived N-terminus peptides have been shown to reduce neutrophil trafficking in several models of acute inflammation [41]. Also human recombinant annexin 1 protects the heart in animals model of myocardial ischemia reperfusion injury[25,83,89]. The protective effect of annexin 1 was associated with reduction in PMNs tissue infiltration, as shown by histological analyses, as well as a parallel reduction in tissue myeloperoxidase activity and reduction in tissue level of pro-inflammatory chemokines and cytokine within the tissue[25,83]. Peptido-mimetics derived from the N-terminal region (Ac2-26 acetyl-AMVSEFLQAWFIENEEQEYVQTVK) have been found to produce similar activity to that of full-length molecule[25,83]. A still open question, however, is that of which receptor mediates the annexin-1 actions and where exactly this is localized on the various cell types. Increasing evidence forward through fMLP or Lipoxin A4 receptor[83,89].

The approach to the PMNs in myocardial infarction, however, should not be limited to the specific activities of the PMN (e.g. activation, migration, infiltration and production of cytotoxic components) but also to the generic inhibition/stimulation of pathways supporting these activities. One of these signaling pathways is that of the phosphoinositide 3-kinases. These are a conserved family of signal transduction enzymes that are involved in regulating cellular activation, inflammatory responses, chemotaxis, and apoptosis[90]. Noteworthy, one of these, the PI3Kγ in neutrophils plays a critical role in the directed-migration of these cells into inflamed tissues. It controls the chemotaxis of these leukocytes by promoting release of phosphatidylinositol (3,4,5)-triphoshate (PIP3), release of Rac and Cdc42, and by F-actin synthesis the reorganization of actin cytoskeleton, important step in cell migration[91]. Data presented here from our group showed that targeted deletion of the PI3Kγ gene in mice generate hearts more sensitive to inflammatory insult and migration of inflammatory components into tissues as it occurs in ischemia/reperfusion procedure. Indeed, in this experimental setting of myocardial infarction the extension of the infarct size as percent of the area at risk and the extension of infarct size as per cent of the cardiac left ventricle is significantly augmented as compared with the wild type mice subjected to the same procedure (Figure 1).

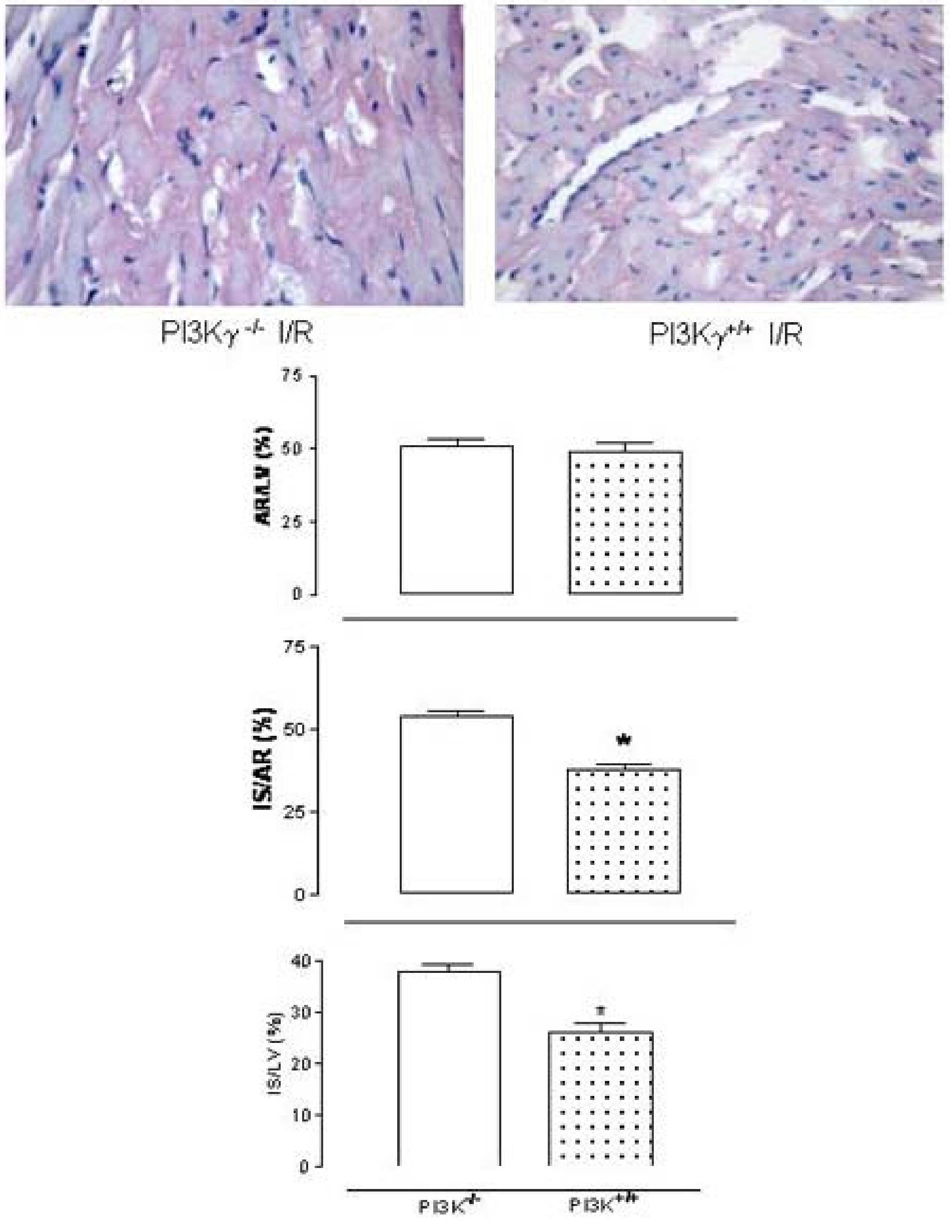

**Figure 1.** The absence of PI3Kγ increases the severity of the myocardial damage induced by ischemia/reperfusion. This was induced by the occlusion of the left anterior descending coronary artery for 25 min followed by 2 h of reperfusion. The infarct size is expressed as a function of the area at risk [25]. PI3Kγ$^{-/-}$ mice and relative wild type littermates were treated intravenously with saline (1 ml/kg). Data are mean ± SE of n=10 mice per group.* P<0.01 vs. respective PI3Kγ$^{-/-}$ mice. IS/AR (Infarct Size/Area at Risk), AR/LV (Area at risk/Left Ventricle) IS/LV (Infarct Size/ Left Ventricle). Panel A) Representative cardiac histological sections; Panel B) Infarct size as calculted.

The damaging effect against the heart injury exerted by the deletion of the PI3Kγ is functionally related to an increased recruitment of blood-borne PMNs into the damaged tissue, as shown by augmentation of local tissue myeloperoxidase activity (Figure 2). The effect is also related to increased production, within the heart tissue, of inflammatory mediators that have been recently coupled to the signalling pathway controlled by the PI3K family of lipid kinases (e.g. cytokines and chemokines)[92] with the end-point being apoptosis and heart damage (Figure 3). Thus, manipulation of the endogenous PI3Kγ signaling may represent a new and novel therapeutic approach to management of the AMI.

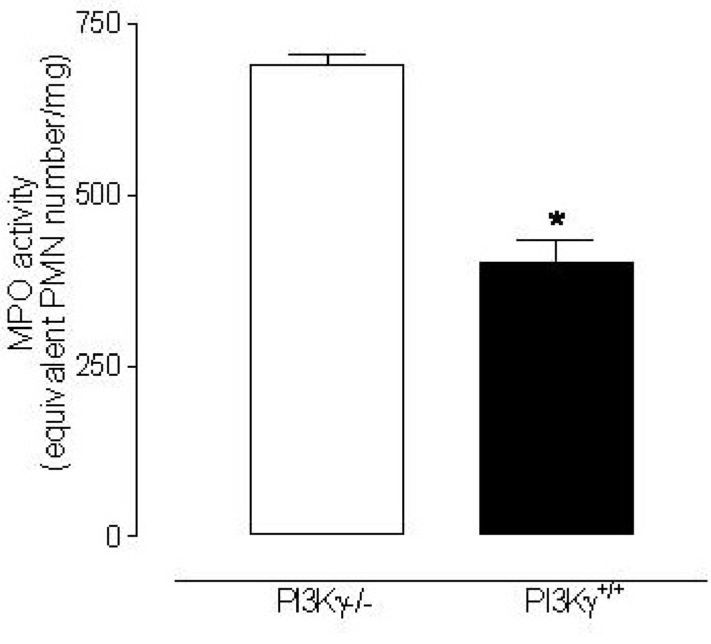

**Figure 2.** The absence of PI3Kγ exacerbates the PMN influx into the myocardium. Myocardial myeloperoxidase activity (MPO, index of PMN tissue infiltration) in infarcted hearts of PI3Kγ$^{+/+}$ and PI3Kγ $^{-/-}$ mice was measured according to the method previously described [25]. Briefly, the area at risk was homogenized in Tris- buffer containing proteinase inhibitors and the homogenates were centrifuged for 30 min at 4,000 x g at 4°C. Aliquot (20 µl) of the supernatant was then allowed to react with a solution of tetra-methyl-benzidine (1.6 mM) and 0.1 mM $H_2O_2$. The rate of change in absorbance was measured with a spectrophotometer at 620 nm. A standard curve was constructed with different numbers of mice neutrophils that were elicited into the peritoneal cavity at 4 h in response to application of 1 ml thioglycollate [25]. Pellets were subjected to three cycles of freeze thawing. Samples (100 µl) were obtained after a final centrifugation at 13,000 r.p.m. for 30 min on a benchfuge. The MPO reaction was produced with a PMN number ranging from 0 to 225 x$10^3$, obtaining a linear relationship with the absorbance at 620 nm following the equation: $A_{620}$=4.504 x no. of PMN + 33.74, with a $R^2$=0.97983. Data of MPO activity are reported as equivalent numbers of PMN per mg of tissue. Data are mean ± SE of n=10 mice per group.* P<0.01 vs. respective PI3Kγ$^{-/-}$ mice.

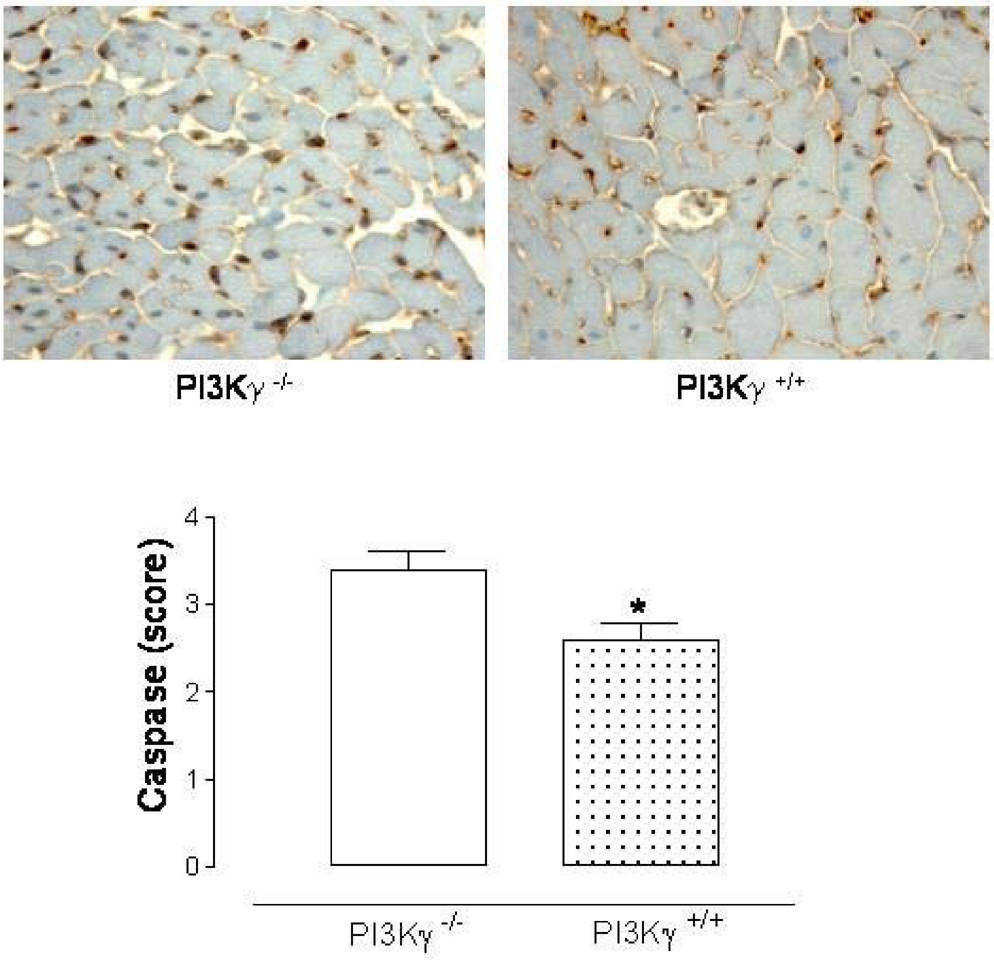

**Figure 3.** Caspase-3 immunostaining (Panel A) and caspase-3 score (Panel B) in infarcted hearts from PI3k$\gamma^{-/-}$ and PI3K$\gamma^{+/+}$ mice. Sections of infarcted myocardial tissue were incubated with anti caspse-3 polyclonal antibody (1:2000 in PBS v/v) and with secondary antibody. In order to confirm that the immunoreaction for the caspase-3 was specific some sections were also incubated with the primary antibody (anti caspase-3). The sections were then scored for intensity of immunostaining (0=absent, 1=faint, 2= moderate, 3=intense) for each antibody, and average value was calculated for each section. *P<0.01 vs PI3K$\gamma^{-/-}$ mice.

Extending the window, a new aspect of this line of research comes up from the studies, done by others first and recently by ourselves, on the role played by the endogenous cannabinoids in AMI. These are an important group of substances newly discovered to be anti-inflammatory mediators[93,94,95,96,97]; they are the active component of *cannabis sativa* and act in part as pan-cytokine inhibitors[94,98,99]. Of the two cannabinoids receptors (CB-R) identified so far the type 2 or CB2-R, is the main peripheral molecular target responsible for the inhibitory properties of the cannabinoids[100,101,102,103,104] have first proposed a pivotal role for the CB2-R, having inhibitory effects on cardiovascular pathologies. They have reported the ability of CB2-R ligands to attenuate myocardial arrhythmias and myocardial damage following I/R. We later added to these pioneer studies the causal link of recruitment of white blood cells to the myocardium as the cause of damage to the heart through activation of CB2-R present on the PMN cell surface, affecting the adhesion/migration of this cell line[105].

On another note, also melanocortins possess cardiac protective activity against experimental I/R exerted through inhibition of the PMNs within the myocardial tissue. This is operated through selective activation of one of the five G protein coupled receptors: the MC3 receptor subtype[106]. To date melanocortin peptides, (e.g. α-melanocyte stimulating hormone, α-MSH) have been shown to possess a multitude of actions including modulation of the host inflammatory response[107] in acute and chronic inflammation, including experimental bowel disease, allergy, and chronic (mycobacterium-induced arthritis) and systemic inflammation (endotoxemia)[108,109]. This inhibitory effect in inflammation has been demonstrated to be due to inhibition of cytokine synthesis and release from target cells *in vitro*[110] and reduction of cytokine levels in inflammatory exudates *in vivo*[107,111]. This anti-inflammatory effect has been shown to occur via inhibition of nuclear transcription factor- kappa B (NF-κB) activation and protection of IκBα degradation[110,112,113,114,115].

An extended paragraph could be that concerning the importance to operate through the reduction of PMN in case of myocardial infarction when this is caused by the insurgence of cardiovascular risk factors such as hypertension, and diabetes. It has been reported that diabetic patients develop congestive heart failure more readily and generally have a greater indication for adverse clinical prognosis and higher mortality. One of the potential mechanisms responsible for the excess mortality in diabetic patients is the greater myocardial injury in response to ischemia/reperfusion[116]. The increased mortality in patients with diabetes in the setting of acute myocardial infarction has been demonstrated in numerous studies, and the mortality after acute myocardial infarction in patients with diabetes is approximately twice that of nondiabetic patients[116].

Many studies have documented that in diabetic patients, micro-macrovascular dysfunction is correlated with changes in blood cell structure and function[117]. These changes in conjunction with changes in the coronary microcirculation have a significant role in the development of diabetic cardiac dysfunction. In fact, abnormal blood-blood vessel interactions in the diabetic coronary microcirculation may have profound effects on the ability of diabetic heart to recover from an ischemic event. Structural and functional abnormalities have been observed in diabetic erythrocytes and leukocytes; particularly, an increase of activated circulating neutrophil leukocytes, and a greater trend to tissue injury mediated by these cell lines. So far we have reported that diabetic rats (streptozotocin-treated rats), submitted to ischemic-reperfusion stimuli, show an increase emigrated leukocytes and a consequent accelerated formation of oxygen reactive species (ROS) within the myocardial tissue, accounting for an enlarged damage[118].

## CONCLUSIONS

The experimental approach thus opens ample opportunities for the study of new therapeutic strategies, or for re-evaluation of the mode of action of already established therapies. The ultimate success of some of these interventions in humans will be itself the proof of the occurrence and relevance of PMN-mediated damage in acute myocardial infarction.

## ACKNOWLEDGMENTS

This review was partly supported by the Italian MIUR (FIRB; n. RBNE01AY9E_006).
